# Grand Challenge—Crossing Borders to Develop Epidemiologic Methods

**DOI:** 10.3389/fepid.2021.786988

**Published:** 2021-11-18

**Authors:** Rolf H. H. Groenwold

**Affiliations:** ^1^Department of Clinical Epidemiology, Leiden University Medical Center, Leiden, Netherlands; ^2^Department of Biomedical Data Sciences, Leiden University Medical Center, Leiden, Netherlands

**Keywords:** bias (epidemiology), bias analysis, prediction, replicability, electronic health record (EHR), statistical analysis

## Introduction

Over the last decades, the epidemiologic landscape has changed dramatically. The amount of data that are currently being made available for epidemiologic research are unprecedented (1). Some argue that the sheer size of, for instance, electronic health records (EHR) databases, in combination with their representativeness of daily clinical practice (the “real world”), carries enormous potential for clinical research (2, 3). Due to open access initiatives, e.g., by funding agencies and journals, datasets are increasingly made available for new collaborative analyses between researchers.

Although the availability of datasets that are both representative of the target population and large (or huge) holds promise in terms of applicability (or generalizability) of research results, at the same time, it still requires valid and accurate methodology. Merely due to the size of many registry-based epidemiologic studies, confidence intervals around effect estimates tend to be very narrow indicating precision. Often, precision is no longer the limiting factor when it comes to applying research results. However, the role of bias can still be considerable: even modest bias can substantially change a study's conclusions (4). Nevertheless, with large datasets, the potential to investigate multiple independent research questions increases, such as on subgroup effects. Yet by studying subgroups of patients in the database, the apparent value of using large datasets is lost, and common epidemiologic challenges become more prominent. Thus, efficient modeling is needed to adequately and appropriately conduct these analyses.

While datasets have become larger, more representative of daily practice, and more accessible to researchers who did not originally collect the data, epidemiologic methods for data collection and data analysis have been refined and are still being improved. Arguably, the development and innovation of new methods occurs within disciplines that seem to become more and more specialized. Below, I discuss different divisions into disciplines and argue that we should look beyond the boundaries of those disciplines to make the next step in developing methods for contemporary epidemiologic research.

## Etiology vs. Prediction

The field of epidemiology has different subfields. While “traditional” epidemiology can be defined as “the investigation of causes of health-related events in populations” (5), which closely links it to health etiology, *clinical epidemiology* appears to be broader by also including research into diagnostic test accuracy, prognosis modeling, and therapeutic interventions (6). Historically, research into methods for etiologic research focused on confounding and other sources of bias, whereas e.g., prediction modeling dealt with optimizing individual risk predictions (7). Although this distinction is artificial and probably too simplistic, it appears to exist in teaching, research, and—importantly—methods development.

Nevertheless, the different subfields can learn from each other, and ideas developed in one subfield can spark developments in another. For example, discussions about estimands (8) and target trial emulation (9) started in research areas with a focus on causality, notably the effects of medical treatments. Nevertheless, these ideas appear worthwhile in a prediction modeling context, too. Similar considerations help to articulate which predictions could be made and how different data analytic strategies potentially lead to predictions with a different meaning (10). Likewise, where measurement error was originally a topic that was deemed relevant only for research of causal effects, recent work suggests that the conceptual framework behind measurement error can be used to understand the impact of variations in measurement procedures when working with prediction models (11). The other way around, penalization and shrinkage methods entered the epidemiological toolbox *via* prediction modeling. Yet, they may also be useful in studies of causal effects, for example, when confronted with a large set of potential confounding variables (12).

## Machine Learning vs. Traditional Statistical Methods

Another distinction within epidemiology that is often made is that between machine learning and “traditional” statistical methods (13). This distinction focuses on the analytical methods that are applied. Yet, the technical aspects are not distinctive; many texts about machine learning start with a description of, for example, linear regression, which is also at the heart of textbooks on (medical) statistics (14). Nevertheless, the more conceptual approach to data analysis may be different between machine learning and “traditional” statistical methods. Where traditional statistical methods focus on testing (pre-specified) hypotheses, machine learning is more open to unraveling (unexpected?) patterns in a dataset. This distinction makes it obvious to resort to traditional statistical methods for etiological research, while machine learning is more popular in prediction modeling research. Nevertheless, data-driven machine learning methods are increasingly applied and could very well have an added value in research into causal effects too (15).

## Replication of Methodological Research

Over the past decades, replicability has increasingly received attention in the social and biomedical sciences, including epidemiology. However, methodological studies seem to lag behind in this development, and statistical simulation studies are no exception. Under strict assumptions and for relatively simple methods, it is possible to mathematically derive how statistical methods will behave when applied to real data, e.g., what type-I error rates will be or to what extent a method can identify an association if it truly exists. However, there are many situations in which this is not the case, for instance, when methods are applied in complex high-dimensional data structures, and when simulation studies may offer a solution (16).

Simulation studies have an important role in guiding the planning of studies and data analysis of applied biomedical research. A striking example is the simulation study by Peduzzi et al. on the sample size required for logistic regression analysis, one of the most used statistical models in the biomedical sciences (17). Since its publication in 1996, this simulation study has had a major impact, has been cited > 6,000 times, and even led to a widely used one-in-ten rule about the number of variables that can be included in a multivariable logistic regression model. However, a replication study by Van Smeden et al. could not confirm these results and questions whether this “one-in-ten rule” is appropriate (18). Undoubtedly, simulation studies are a powerful tool for methodological research, yet this example illustrates that they may have limitations too and that their results are clearly not definitive. Instead of considering the results of simulation studies to be set in stone, simulation studies need to be replicated, just as empirical studies need replication (19).

## Crossing Borders

In parallel with spectacular developments in terms of the dimension of datasets available for epidemiological research, we have witnessed developments regarding the methods being applied to analyse those data. Even though the abovementioned distinctions may appear a bit artificial, they also illustrate that looking for appropriate methodology outside of one's own field may prove worthwhile. When doing so, we need to keep an eye out on the appropriateness of those methods and the fundamental questions about whether (and how) the new methods still provide correct and interpretable answers to the research questions that are being asked. Testing and refining methods and discussing their suitability within a new field of application is obviously essential. Often innovations come from adjacent fields, and why would epidemiology be an exception?

The section *Research Methods and Advances in Epidemiology* aims to contribute to the development of new and existing epidemiologic methods and to help researchers cross the borders that have emerged between epidemiological fields. This section of Frontiers in Epidemiology seeks not only original research describing new methods, but will also seek challenges of the use of existing methods for modern epidemiology research questions, as well as replication of already developed analytical methods for such research questions.

## Author Contributions

The author confirms being the sole contributor of this work and has approved it for publication.

## Conflict of Interest

The author declares that the research was conducted in the absence of any commercial or financial relationships that could be construed as a potential conflict of interest.

## Publisher's Note

All claims expressed in this article are solely those of the authors and do not necessarily represent those of their affiliated organizations, or those of the publisher, the editors and the reviewers. Any product that may be evaluated in this article, or claim that may be made by its manufacturer, is not guaranteed or endorsed by the publisher.
